# Primary vulval sebaceous carcinoma: rare case report and literature review

**DOI:** 10.3389/fonc.2025.1585840

**Published:** 2025-08-20

**Authors:** Kaige Pei, Jiawen Zhang, Mingrong Xi

**Affiliations:** ^1^ Department of Gynecology and Obstetrics, West China Second University Hospital, Sichuan University, Chengdu, Sichuan, China; ^2^ Key Laboratory of Birth Defects and Related Diseases of Women and Children, Sichuan University, Chengdu, Sichuan, China

**Keywords:** vulval sebaceous carcinoma, sentinel lymph node biopsy, extraocular sebaceous carcinoma, rare case report, literature review

## Abstract

This study presents a rare case of primary vulval sebaceous carcinoma (SC) and a literature review. Sebaceous carcinoma is an aggressive skin malignancy, predominantly periocular, with extraocular cases being particularly uncommon. We detail a 50-year-old female patient diagnosed with primary vulval SC, exhibiting a 0.5 cm white lesion on her left vulva. Despite initial drug therapy, the lesion progressed to ulceration and induration. Histopathological examination confirmed SC. The patient underwent extensive local vulvectomy and sentinel lymph node biopsy, showing no evidence of metastasis. At 22 months post-surgery, the patient remained recurrence-free. A literature review identified 13 additional cases, highlighting diverse presentations and management strategies. Our findings underscore the importance of sentinel lymph node biopsy and tailored surgical approaches for early-stage disease. The study contributes to the understanding of this rare condition and may inform future management protocols.

## 1 Introduction

Sebaceous carcinoma (SC) is a rare but potentially aggressive cutaneous malignancy, predominantly occurring in the periocular region, with fewer cases reported in extraocular sites such as the face, scalp, trunk, limbs, and vulva (1). Accounting for less than 5% of all cutaneous malignancies (2), SC is categorized into two subtypes based on location: periocular and extraocular SC, with approximately one-third to three-fourths classified as periocular (3). Over 90% of extraocular SC are located in the head and neck region, with only 7.2% originating in other areas (4).

Primary vulval SC is an extremely rare form of extraocular SC, with only a few cases reported in the literature (5). There is currently no standardized approach to the clinical features and management strategies for this rare disease, which are primarily based on experiences from individual case reports. Clinically, patients may present with localized erythema (6), cysts (7), papules (8, 9), ulcers (10), yellowish-white nodules (11), sclerotic plaques (12), or exophytic tumors (13). In terms of management, surgical approaches for the primary lesion have included radical vulvectomy, extensive local vulvectomy, and simple excisional biopsy (7–9, 11, 14). The necessity of inguinal lymphadenectomy remains controversial (3, 14), with sentinel lymph node biopsy (SLNB) proposed as an alternative to avoid undertreatment and reduce complications associated with unnecessary lymphadenectomy (14). Postoperative chemotherapy and radiation have been reported, but their benefits remain unclear (1, 6, 11). Given the limited understanding of this rare disease, further exploration of its clinical features and management strategies is essential.

This article details the diagnosis and treatment of a patient with primary vulval SC at our institution and reviews previously reported cases and relevant literature to contribute to the understanding of this rare condition.

## 2 Case report

A 50-year-old woman was admitted to the dermatology department of a local hospital due to the discovery of a 0.5-cm white growth on her left vulva. Considering the inflammatory lesions, no significant improvement was observed after drug treatment. Therefore, the patient spontaneously burst the growth, and then ulcers and induration appeared in the site, which did not heal for a long time. The patient was treated again in the dermatology department of a local hospital and underwent local lesion excision biopsy. The pathological results indicated SC, and the patient was referred to our hospital. The woman was generally in good condition and had no previous history of cervical and endometrial lesions, intestinal polyps, or colorectal cancer, and no family history of malignant tumors.

**Figure 1:**
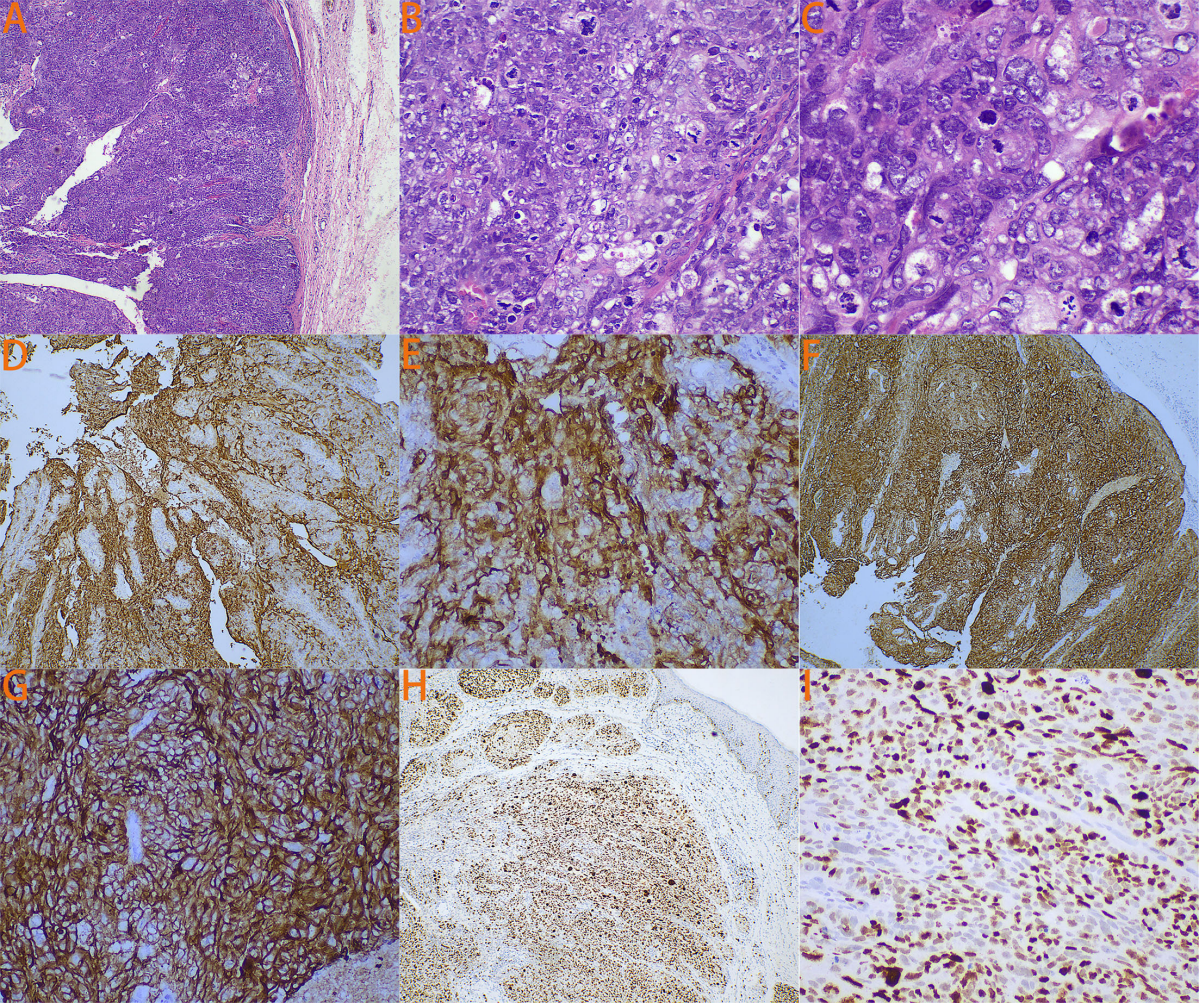
The patient’s detailed pathological features. **(A)** Under 4x magnification, it is possible to see cancer cells forming irregular nest-like structures. **(B)** Under 20x magnification, the cancer cells exhibit large nuclei with prominent nucleoli, and mitotic figures are commonly visible. Additionally, the cytoplasm is abundant, and vacuolar structures can be observed within the cytoplasm of some cells. **(C)** Under 40x magnification, it can be observed that the cancer cells have poorly developed nuclei with unevenly distributed nuclear chromatin and irregular nuclear membranes. Some cells contain a large amount of lipids. **(D)** Immunohistochemical staining for EMA under 4x magnification. **(E)** Immunohistochemical staining for EMA under 20x magnification. **(F)** Immunohistochemical staining for PANCK under 4x magnification. **(G)** Immunohistochemical staining for PANCK under 20x magnification. **(H)** Immunohistochemical staining for Ki67 under 4x magnification. **(I)** Immunohistochemical staining for Ki67 under 20x magnification.

The patient’s detailed pathological features are shown in **Figure 1**. At low magnification (**Figure 1A**), irregular nest-like structures formed by cancer cells are visible, with some areas showing a pronounced infiltrative growth pattern, where cancer cell nests invade the surrounding stroma. At medium magnification (**Figure 1B**), the cancer cells exhibit large nuclei with prominent nucleoli, and mitotic figures are commonly seen, indicating the high degree of anaplasia and proliferative activity of the tumor cells. The cytoplasm is abundant, and vacuolated structures can be observed within the cytoplasm of some cells, suggesting sebaceous gland differentiation features. At high magnification (**Figure 1C**), significant nuclear anaplasia is apparent, with uneven distribution of nuclear chromatin and irregular nuclear membranes. Some cells contain a large amount of lipid, which is a characteristic manifestation of SC. Immunohistochemistry shows positive staining of tumor cells for EMA (**Figures 1D, E**), PANCK (**Figures 1F, G**), with a Ki-67 index of approximately 70% (**Figures 1H, I**). HMB45, Melan-A, and S-100 are negative. Among these, EMA is an important marker for SC. The expression pattern of PANCK aids in distinguishing adenocarcinoma from other types of cancer, such as squamous cell carcinoma and undifferentiated carcinoma. SC typically presents with a higher Ki-67 index. Based on morphological and immunohistochemical findings, the diagnosis of vulval SC was favored.

**Figure 2:**
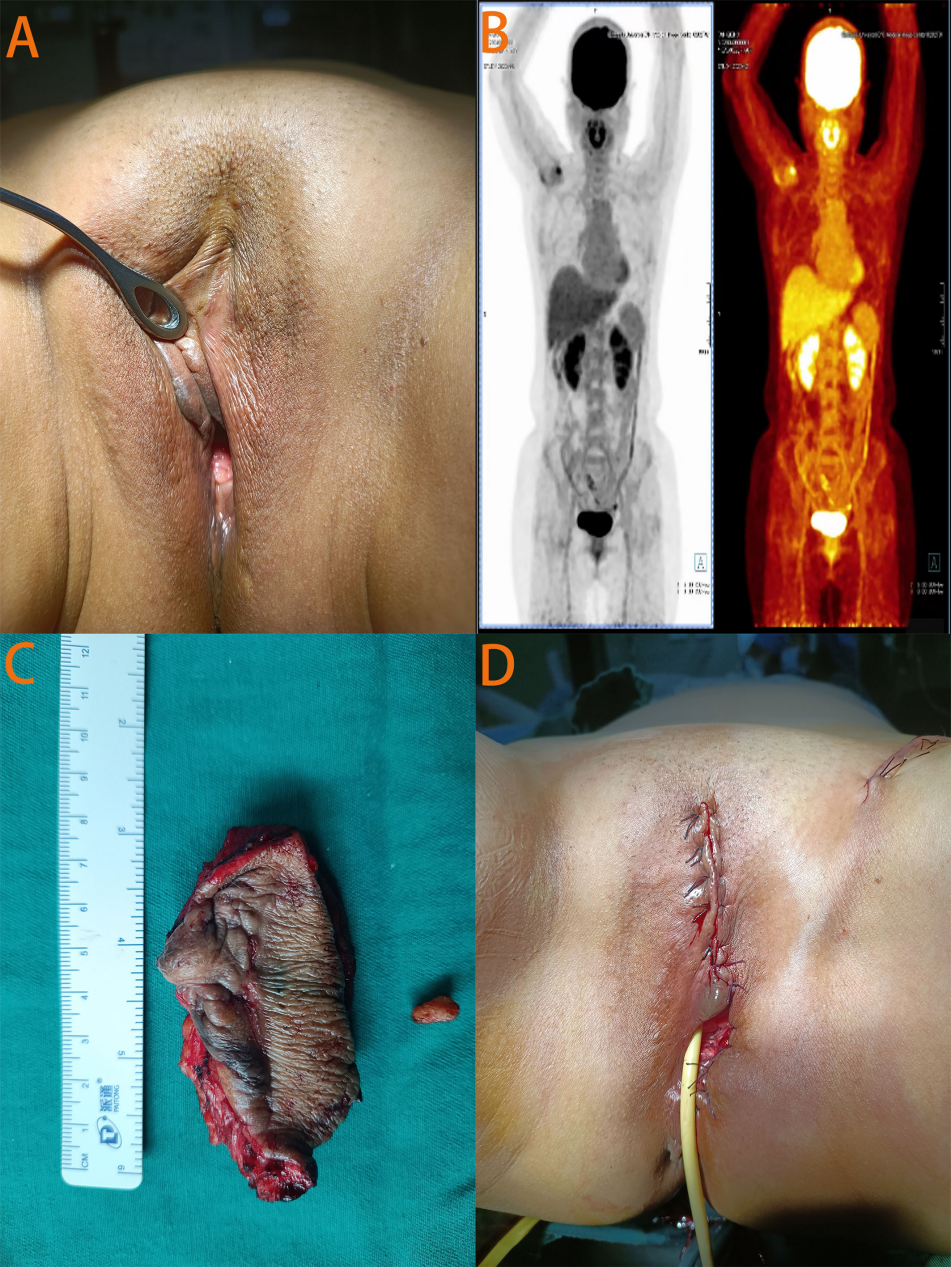
Relevant auxiliary examination images and pre - and post-operative photos of the patient. **(A)** In the photo taken before surgery, a scar about 1cm in size was visible at the primary site of the disease. **(B)** The PET-CT images showed no evidence of disease elsewhere. **(C)** The surgically excised left vulvar tissue and left inguinal lymph node. **(D)** Photograph taken at the end of the surgery.

On physical examination, the patient had rough skin on the upper one-third of the left labia majora with a 1-cm surgical scar (**Figure 2A**). No masses were palpable around the scar. Routine gynecologic ultrasound revealed several intramural and subserosal uterine fibroids, with the largest measuring 1.5 cm in diameter; no other abnormalities were noted. To rule out other primary tumors and assess for local and distant metastasis, Positron Emission Tomography combined with Computed Tomography (PET-CT) was performed, showing no evidence of tumor elsewhere (**Figure 2B**). After reviewing the literature and thorough discussion, the patient underwent local extensive vulvectomy (**Figures 2C, D**). Intraoperatively, nanocarbon sentinel lymph node mapping was performed in the left inguinal region. No sentinel lymph nodes were visualized, but a 1-cm enlarged lymph node was palpated and subsequently excised. Postoperative pathology revealed no evidence of carcinoma in the excised vulvar tissue or left inguinal lymph node. The patient had an uneventful recovery with no infection or lymphedema, and the incision healed perfectly. At 22 months of follow-up, the patient remained asymptomatic with no signs of recurrence.

## 3 Literature review

In order to enroll all the suitable vulval SC patients reported as of February 15, 2025, we used the following keywords to search in major medical databases (PubMed, Embase, Web of Science, and Scopus): “vulval sebaceous carcinoma”; “sebaceous carcinoma of the vulva”; “primary vulval sebaceous carcinoma”; “sebaceous carcinoma of female genital tract”; “extraocular sebaceous carcinoma.” All the related articles of vulval SC cited by the screened papers would have been also evaluated to determine whether they were eligible. Patients who were diagnosed as extraocular SC of other sites or secondary vulval SC were excluded from final analysis. Similarly, those who lacked detailed clinical characteristics were also eliminated. To ensure the scientific nature and reliability, we excluded vulval SC patients from letter to editors and non-English publications. Moreover, unrelated articles, including imaging studies and or pathological investigations of vulval SC were not subjected to analysis. The **Supplementary Figure S1** shows the screening process of our research. We eventually included 14 patients (**Table 1**) with vulval SC, including 13 patients identified in literature review and the one patient treated in our hospital.

**Table 1:** Cases of sebaceous carcinoma of vulva reported in the literature.

References	Year	Age	Lesion	Size	Appearance	Duration of symptoms	Treatment	Metastasis	Follow-up	Outcome
Jacobs et al (15).	1986	89	Left labia minora	Two lesions: 3.0 × 1.4 cm and 1.0 × 0.8 cm.	Pink white plaque	1 year	Left radical hemivulvectomy	None	N/A	N/A
Kawamoto et al (11).	1995	78	Left labia minora	2.5 × 1.5 cm	Yellow white nodule	6 months	Simple vulvectomy Inguinal lymphadenectomy Radiotherapy (left inguinal area)	Left inguinal lymph nodes	17 months	NED
Carlson et al (7).	1996	46	Left labia majora	Not stated	Cyst	Not stated	Left radical hemivulvectomy Left inguinal lymphadenectomy	None	31 months	NED
Escalonilla et al (13).	1999	76	Right labia majora	4 cm × 3 cm	Red white tumor and small papule	4 months	Radical vulvectomy Inguinal lymphadenectomy	None	12 months	NED
Khan et al (1).	2003	49	Right labia minora	0.5 cm	Papilloma	Not stated	Local excision Inguinal lymphadenectomy Radiotherapy	Left inguinal lymph node	11 months	AWD (recurrence after 7 months)
Pusiol et al (16).	2011	51	Left labia majora	2.5 × 1.5 cm	Exophytic red and white tumor	6 months	Hemivulvectomy	None	18 months	NED
Sullivan et al (8).	2016	76	Left vulva	0.5 cm	Visible papule	Not stated	Local excision Left inguinal lymphadenectomy	None	10 months	NED
Thakur et al (10).	2017	55	Right labia majora and minora	Two lesions: 2.5 × 2 cm and 1 × 1.5 cm	Ulcerative nodule	4 months	Radiotherapy	Right inguinal lymph node	18 months	NED
Hind Alharthi et al (6).	2021	27	Left labia majora	1 cm	Red tender firm lesion Painful vaginal swelling	4 weeks	Chemotherapy	Vagina Pelvic Lung Left ischium Para-aortic and inguinal lymph nodes	8 months	DOD
Ayaka Yamamoto et al (14).	2021	66	Left labia minora	8 mm	Ulcerated lesion	Not stated	Local excision Sentinel lymph node biopsy	None	14 months	NED
Ana Carolina Rocha et al (9).	2022	78	Right labia majora	12 x 10 mm	Yellow papule	Not stated	Partial right vulvectomy Right inguinal sentinel node biopsy	None	12 months	NED
Xiaoxue Wang et al (5).	2023	62	The surface of the left labium and mons pubis	The largest diameter of about 2.5 cm	Multiple erythemas	3 years	Radical wide local excision	None	12 months	NED
Xiaoxue Wang et al (5).	2023	31	Left labia majora	0.5 cm	Mild painful nodule with slight itching	2 years	Radical wide local excision	None	49 months	NED
Present case	2025	50	Left labia majora	0.5 cm	White lesion Ulcers and indurations	1 month	Local extensive excision of vulva Left inguinal sentinel node biopsy	None	22 months	NED

N/A, not available; NED, no evidence of disease; AWD, alive with disease; DOD, died of disease.

The 14 patients ranged in age from 27 to 89 years, with an average age of 59.6 years. Only two patients were under 45 years old, indicating that the disease predominantly affects middle-aged and elderly individuals. Among the 14 patients, 10 had tumors originating on the left vulva, while the remaining four had tumors on the right vulva. The largest primary tumor measured 4 cm, while the smallest was 0.5 cm, with an average size of approximately 1.6 cm. As shown in **Table 1**, clinical presentations of vulval SC are diverse, including plaques, nodules, cysts, papules, ulcers, and exophytic tumors, with colors ranging from pink to yellow, red, and white. These symptoms persisted from one month to three years.

Twelve of the 14 patients (85.7%) underwent surgical treatment, with various surgical options including excisional biopsy, local excision, extensive local excision, radical hemivulvectomy, and radical vulvectomy. Among the 12 surgical patients, five (41.7%) also underwent inguinal lymphadenectomy, with two (40%) showing lymph node involvement. Three patients (25%) underwent inguinal lymph node biopsy, with no lymph node involvement detected. Most patients (10/12, 83.3%) did not receive any postoperative adjuvant therapy, except for two patients with inguinal lymph node metastasis who received postoperative radiation therapy. Among the two patients who did not undergo surgery, one had chronic kidney disease with a concurrent urinary tract infection and was referred to a cancer treatment center for radiation therapy. The other patient had pelvic and pulmonary metastases at initial diagnosis, was diagnosed with stage IV SC, and was treated with a chemotherapy regimen consisting of carboplatin and paclitaxel. Unfortunately, the chemotherapy was not effective, and the patient passed away eight months after initial diagnosis.

The median follow-up time for these patients was 14 months (range: 8–49 months). At the last follow-up, 11 patients had no evidence of disease, one patient had the disease, and one patient had died from the disease. The 3-year disease-specific survival rate (DSS) for these patients was 92.3% and the 3-year recurrence-free survival rate (RFS) was 84.6%.

## 4 Discussion

SC is a rare but potentially aggressive cutaneous malignancy that commonly occurs in the eyelids, known as periocular SC, while extraocular SC primarily affects the head, neck, and trunk (3, 17). Despite the presence of numerous sebaceous glands in the vulva, primary vulval SC is extremely rare and clinically diverse. Due to its rarity, other differential diagnoses must be excluded, including primary and metastatic skin tumors such as basal cell carcinoma with sebaceous differentiation, squamous cell carcinoma with clear cell features, melanoma, and metastatic carcinoma from other sites (6). Immunohistochemistry aids in distinguishing these conditions. Basal cell carcinoma typically tests negative for BerEp4 and EMA. Squamous cell carcinoma usually stains positive for CK5/6, P63, and negative for EMA. Melanoma commonly demonstrates positivity for S100, HMB45, Melan-A, and negative for EMA. Metastatic adenocarcinoma generally shows positivity for CK7, CK20, and negativity for EMA.

Vulval SC, a rare vulval cancer subtype, should follow general vulval cancer treatment guidelines. Despite diverse guidelines with different versions, their diagnostic and treatment principles are largely the same (18). During diagnosis, the initial clinical examination must accurately record the anatomical extent of the lesion, such as involvement of the labia minora and/or labia majora, clitoris, urethra, anus, or perineum, as these details are crucial for pathological assessment. All guidelines agree that biopsies are needed for all suspected vulval lesions to confirm the diagnosis, with each lesion carefully localized and fully described. For imaging to aid diagnosis, options include inguinal femoral lymph node (IFLN) ultrasound, Computed tomography (CT), magnetic resonance imaging (MRI), or 18F-fluorodeoxyglucose positron emission tomography/computed tomography (18F-FDG-PET/CT). In terms of treatment, for early-stage vulval cancer, surgical approaches vary based on tumor size, depth of invasion, lymphovascular space invasion (LVSI) presence, and tumor location. Procedures range from wide local excision with complete IFLN dissection to individualized tumor removal (simple partial vulvectomy, radical partial vulvectomy, or radical local excision) plus SLNB. For IFLNs, staging methods are chosen based on involvement risk, such as IFLN ultrasound, SLNB, or IFLN dissection. For advanced vulval cancer, patients with unresectable lesions have radical radiotherapy as the first choice. Even if the lesion is resectable but with lymph node or distant metastasis, radiotherapy should be added, along with chemotherapy if needed. Adjuvant therapy depends on local risk factors. If there’s one or more risk factors like positive margins, extensive LVSI or perineural invasion, large tumor (> 4cm), multiple lesions, stromal invasion > 5mm, or lymph node involvement, adjuvant therapy should be considered (18).

Surgery is the recommended first-line treatment for both periocular and extraocular SC (3). Local treatment for SC often involves complete circumferential peripheral and deep margin assessment or Mohs micrographic surgery, in which surgical excision is followed by pathologist-rendered strict margin assessment using rapid frozen section examination (19). Previous studies have shown that radical vulvectomy inevitably increases the psychosexual morbidity associated with treatment (5). In this case, given the very small size of the tumor, extensive local vulvectomy was performed after careful discussion, minimizing the patient’s vulvar appearance change and preserving sexual function without compromising treatment efficacy.

SLNB has been shown to be beneficial in the treatment of periocular SC (20, 21). However, its value in the treatment of extraocular SC requires further validation (3). For vulval SC, on one hand, treatment must refer to vulval cancer, for which SLNB is meaningful (22). On the other hand, existing studies have shown that the incidence of lymph node involvement in vulval SC is as high as 50% (5), much higher than the 0.9% in extraocular SC (23), possibly due to the extensive lymphatic circulation in the perineal area. Therefore, we believe that inguinal lymphadenectomy cannot be unconditionally omitted for patients with vulval SC. To avoid missing positive lymph nodes and reducing the side effects of lymphadenectomy, SLNB has increasingly been used in vulval and extraocular SC (5). Comprehensive imaging, preoperative evaluation and routine SLNB may be feasible for early vulval SC. Thus, in our case, nanocarbon sentinel lymph node mapping was performed, and the suspicious lymph node was excised for biopsy.

Given the extreme rarity of vulval SC, there is no evidence to suggest that radiation therapy and chemotherapy improve prognosis. However, in clinical practice, for patients with vulval SC who are medically unfit for surgery, have positive surgical margins, or have lymph node metastasis, radiation therapy and chemotherapy are often used as first-line and adjuvant treatments (1, 6, 10, 11). Particularly, drawing on the therapeutic experience of vulval cancer, post - operative adjuvant therapy (radiotherapy, chemotherapy, or chemoradiotherapy) is beneficial for vulval SC patients with positive surgical margins, inguinal lymph node metastasis, or severe LVSI. In vulval cancer, the GROINSS-V-II trial found that inguinal radiation therapy is a safe alternative to inguinal lymphadenectomy for patients with sentinel lymph node micrometastases (24), indirectly supporting the use of radiation therapy as adjuvant treatment for vulval SC. Among all previously reported cases of vulval SC, only one patient received a chemotherapy regimen consisting of carboplatin and paclitaxel for eight cycles, but the treatment was not effective, and the patient died eight months after initial diagnosis. However, due to the small sample size, this does not indicate that vulval SC is not sensitive to chemotherapy. Further research is needed on the types, doses, and duration of chemotherapy to achieve better outcomes in treating vulval SC. For unresectable extraocular SC, patients may benefit from a multimodal approach. Surgical resection with negative pathologic margins is the mainstay treatment. Preoperative systemic workup, including radiographic imaging or SLNB, may be warranted. Adjuvant radiation therapy can be considered for recurrent and metastatic tumors (25).

When managing patients with SC, the possibility of Muir-Torre syndrome (MTS) must always be considered. Characterized by the presence of sebaceous tumors and one or more visceral malignancies, MTS includes solitary or multiple sebaceous adenomas and/or carcinomas, with colorectal cancer and endometrial cancer being the most common visceral malignancies (25, 26). Some patients with MTS also have germline mutations in the DNA mismatch repair genes MLH1 or MSH2, considered a subtype of Lynch syndrome (LS) (27). Given LS overlaps with MTS partly, screening via immunohistochemistry for MSH2/MLH1/PMS2 is now recommended in many centers and genetic screening be paid more attention. Research showed that SC may be associated with the cancer predisposition syndrome (MTS/LS), identifiable by SC mismatch repair (MMR) screening. Incorporation of MMR screening into clinical practice guidelines for the management of SC will increase the opportunity for MTS/LS diagnoses, with implications for cancer surveillance and immunotherapy treatment targeted to MTS/LS cancers (28). MTS is extremely rare (29), and routine screening of all SC patients would be cost-ineffective in terms of health care. Therefore, the Mayo MTS risk score system was established to identify patients who need further evaluation for MTS (30). The score system includes four variables: age, number of sebaceous tumors, and personal and family history of Lynch syndrome-related cancers. For patients with a score ≥2, the sensitivity and specificity for identifying MTS are 70% and 98%, respectively (30). In this case, the patient had no family or personal history, and PET-CT imaging showed no evidence of other malignancies, ruling out MTS.

In summary, patients with vulval SC have a favorable prognosis, with a 3-year DSS of 92.3% and a 3-year RFS of 84.6%. For patients with early-stage vulval SC, radical local extensive excision is the preferred treatment, and SLNB is recommended. Postoperative adjuvant therapy can be avoided in patients with negative surgical margins and no lymph node involvement. When positive margins or metastatic disease is present, treatment of vulval SC can refer to vulval cancer guidelines. Additionally, screening for MTS in patients with vulval SC should be emphasized, especially in younger patients. However, due to the extreme rarity of vulval SC, its clinical presentation, optimal treatment strategy, and prognosis require further evaluation.

## Data Availability

The original contributions presented in the study are included in the article/**Supplementary Material**. Further inquiries can be directed to the corresponding author.
