# Supplementary figures and images for: Primary vulval sebaceous carcinoma: rare case report and literature review
Source: Front Oncol. 2025 Aug 20;15:1585840. doi: 10.3389/fonc.2025.1585840 (PMC12404968; doi:10.3389/fonc.2025.1585840)

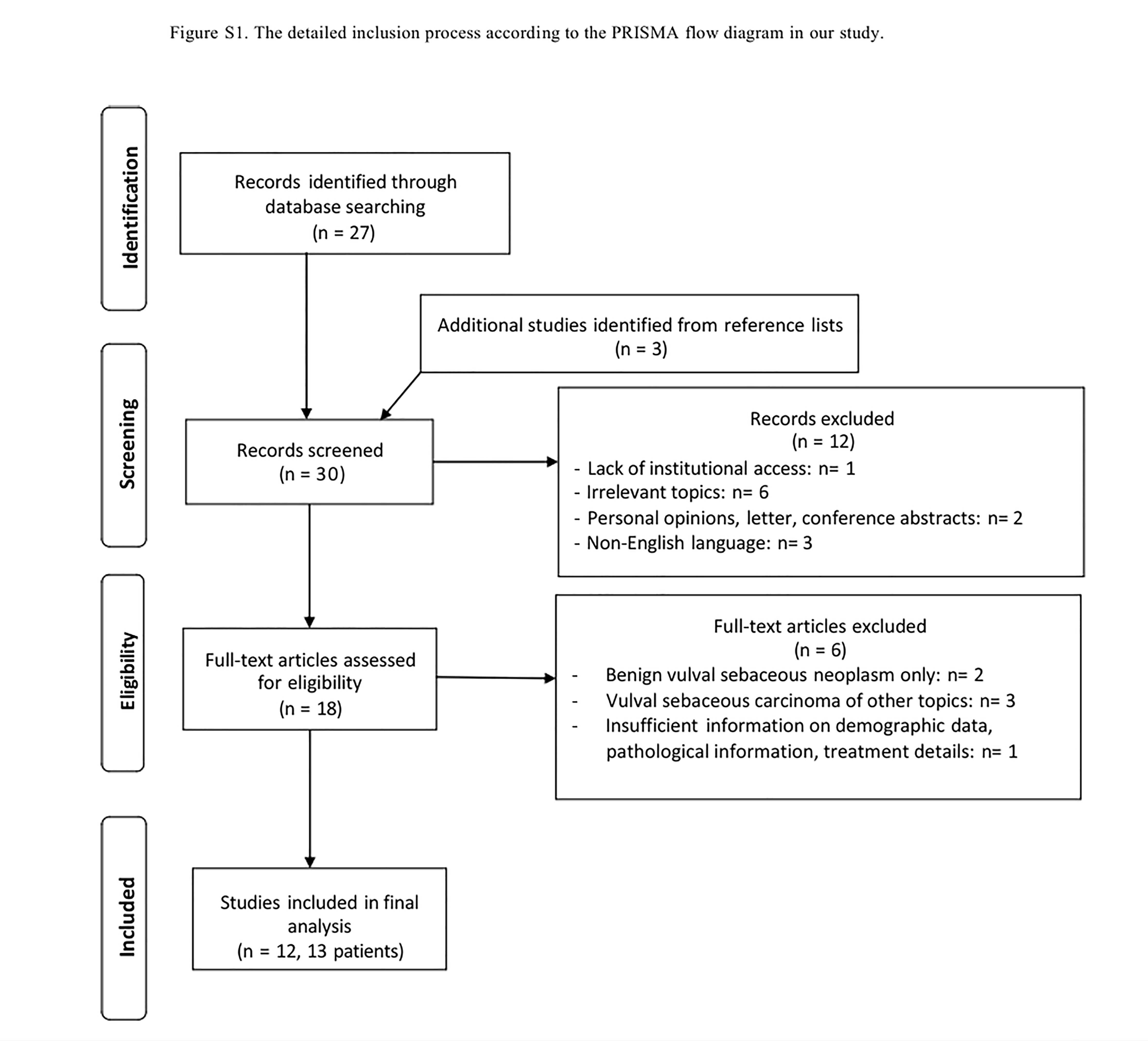

Supplement: Supplementary file 1 [file Image1.tif]
